# Biochemical Parameters for Longitudinal Monitoring of Liver Function in Rat Models of Partial Hepatectomy Following Liver Injury

**DOI:** 10.1371/journal.pone.0066383

**Published:** 2013-06-18

**Authors:** Nele Boeykens, Peter Ponsaerts, Dirk Ysebaert, Kathleen De Greef

**Affiliations:** 1 Laboratory of Experimental Surgery, Antwerp Surgical Training and Research Center, University of Antwerp/University Hospital of Antwerp, Antwerp, Belgium; 2 Laboratory of Experimental Hematology, Vaccine and Infectious Disease Institute (Vaxinfectio), University of Antwerp, Antwerp, Belgium; National Cancer Institute, United States of America

## Abstract

**Background:**

While evaluation of liver function in preclinical animal studies is commonly performed at selected time-points by invasive determination of the liver/body weight ratio and histological analyses, the validation of longitudinal measurement tools for monitoring liver function are of major interest.

**Aims:**

To longitudinally evaluate serum cholinesterase (CHE) and total serum bilirubin (TSB) levels as non-invasive markers to determine injury- and partial hepatectomy (PHx)-induced alterations of liver function in rats.

**Methods:**

Male and female Lewis rats were subjected to either methionine/choline deficient (MCD) diet or treatment with FOLFOX chemotherapy prior to PHx. Body weight and CHE/TSB levels are determined weekly. Following PHx and at the study end, histological analyses of liver tissue are performed.

**Results:**

Following MCD diet, but not after FOLFOX chemotherapy treatment, results indicate gender-specific alterations in serum CHE levels and gender-independent alterations in TSB levels. Likewise, histological analyses of resected liver parts indicate significant liver injury following MCD-diet, but not following FOLFOX treatment. While TSB levels rapidly recover following MCD diet/FOLFOX treatment combined with a PHx, serum CHE levels are subject to significant model- and gender-specific differences, despite full histopathological recovery of liver tissue.

**Conclusions:**

Longitudinal measurements of serum CHE levels and TSB levels in rats are highly complementary as non-invasive parameters for evaluation of liver injury and/or recovery.

## Introduction

Evaluation of induced liver injury and subsequent liver regeneration in rodents is most commonly performed on the basis of imaging techniques (MRI/CT), histological analyses, determination of the body/liver weight ratio and evaluation of liver function parameters at selected time-points. However, in order to accurately determine the effect of therapeutic interventions during pre-clinical studies, longitudinal assessment of liver function is imperative to identify long-term functional deficits or impaired/improved regeneration. For this purpose, weekly analyses of serum cholinesterase (CHE) and total serum bilirubin (TSB) levels may provide us with more information as compared to selected time-point analysis for monitoring liver function in preclinical research on liver injury and/or regeneration. In this study, we focus on the long-term assessment of liver function in two rat models of partial hepatectomy (PHx) following liver injury.

With a prevalence of 10–20% in lean population and 50–75% in obese population, steatosis is the most common chronic liver disease worldwide [1]. In some patients, hepatic steatosis may progress to non-alcoholic steatohepatitis (NASH), cirrhosis and even develop hepatocellular carcinoma. Current clinical observations suggest an increased risk of performing PHx in patients with severe steatosis [2], [3], [4]. Moreover, experimental studies have reported impaired liver regeneration in animal models of steatosis [5], [6]. A methionine/choline deficient (MCD) diet has been established to mimic pathological conditions of hepatic steatosis and is frequently used in preclinical research [1]. Therefore, in this study we longitudinally evaluate liver function in rats during MCD diet (i.e. the induction of human NASH-like pathology) and during the recovery phase following PHx.

Chemotherapy associated steatohepatitis (CASH) and also sinusoidal obstruction syndrome (SOS) have clinically been observed in patients subjected to preoperative chemotherapy treatment in order to downstage non-resectable liver tumors and/or colorectal liver metastases (CRLM) [7], [8], [9], [10]. Thereby it is suggested that the type and severity of liver injury following preoperative chemotherapy is correlated with the type of chemotherapy regimen [8], [11]. As the pathogenesis of CASH and SOS is currently poorly understood, the development of an experimental animal model to study both consequences is needed. Simultaneously to the above-described model for NASH, in this study we longitudinally evaluate liver function in rats subjected to the commonly used clinical FOLFOX chemotherapy treatment for CRLM. Subsequently, liver recovery following PHx of CASH livers is monitored longitudinally.

Finally, gender-dependent differences in response rate, probability of side effects and toxicity in patients treated with chemotherapy have been suggested [12], [13], [14]. For example, as the half-life of several anti-cancer drugs is longer in women, which is related with enhanced survival, an increased toxicity can be observed [15]. Therefore, our proposed studies are identically performed in both male and female rats in order to detect gender-specific differences.

In summary, in this study we present the longitudinal assessment of liver function, based on serum CHE and TSB levels, in both male and female rats following liver injury, as well as during subsequent recovery after PHx. At selected time points, i.e. after PHx and at the study end, histological analyses of liver tissue are performed.

## Materials and Methods

### Animals

Male and female wild type Lewis rats (weighing 115–125 g, n = 80) were obtained via Charles River Laboratories (strain code 004). Rats were kept in normal day-night cycle (12/12) with access to food and water *ad libitum*. All experimental procedures were approved by the Ethical Committee for Animal Experiments of the University of Antwerp (approval no. 2008/19).

### Methionine/choline-deficient (MCD) Diet

Rats were subjected to a methionine/choline-deficient (MCD) diet (Harlan Laboratories, Inc.) *ad libitum* for 4 weeks, resulting in liver injury comparable to human steatotic liver, as assessed by phenotypic, laboratory and histological parameters [16].

### Chemotherapy

According the FOLFOX scheme, which is commonly used for treatment of colorectal liver metastases [17], 5-fluoro-uracil (5-FU) (27 mg/kg, Fluracedyl®), oxaliplatin (2,3 mg/kg, Eloxatin®) and leucovorin (2,7 mg/kg, Elvorine®) were administered weekly for 10 weeks. Rat doses were calculated according to the human dose via the following conversion formula (37 mg/m^2^ human dose = 1 mg/kg rat dose; [18]). All drugs were prepared immediately before use, by dilution in 0,9% NaCl (5-FU and leucovorin) or glucose (oxaliplatin) and injected subcutaneously in a volume of 100–200 µL (for each drug) according to the body weight. All chemotherapeutics were obtained from the pharmacy of the Antwerp University Hospital (UZA). Control animals received subcutaneous injections of glucose and 0,9% NaCl according to the body weight.

### Partial Hepatectomy (PHx)

PHx (70%) of rat liver was performed as previously described by Mitchell and Willenbring [19], with minor modifications. Briefly, general gas anesthesia was induced and maintained by a mixture of O_2_ and N_2_O (0.5 l/min and 1.5 l/min) and isoflurane (Isoflo®, 4% for induction and 2% for maintenance). All interventions were performed under sterile conditions, while body temperature was kept on 37°C using a heating pad with feedback control by an intrarectally placed sensor. For PHx, the abdomen was opened via a midline skin and muscle incision. The median and left lateral lobe were ligated and resected. The resected liver specimen was immediately weighed in order to estimate liver mass. After PHx, 1 ml 0,9% NaCl was given intraperitoneally and the abdomen was closed with a silk running suture. Next, rats were allowed to recover from anesthesia under heat-producing lamps.

### Peripheral Blood Serum Analysis

Peripheral blood was taken weekly via the tail vein (600 µl) and collected in 600 µl multivette tubes (Sarstedt). Following blood clotting (30 min.), samples were centrifuged at 1500 g for 10 minutes. Next, serum was collected and stored at −20°C until analysis. Serum cholinesterase (CHE), total serum bilirubin (TSB), alkaline phosphatase (AP), creatinin (Cr), glutamic oxaloacetic transaminase (GOT) and glutamic pyruvic transaminase (GPT) were determined in duplicate by the central laboratory of the Antwerp University Hospital (UZA) using a Dimension Vista 1500 Intelligent Lab System (Siemens).

### Histological Analysis

Following PHx and euthanasia by heart puncture at the end of the study, the resected liver parts or the (regenerated) livers were removed and directly weighted in order to calculate the level of liver regeneration in weight (see below). Next, (partial) livers were divided in two and fixed in methacarn or in formol/calcium. Following fixation in methacarn (60% methanol, 10% acetic acid, 30% trichlorethane) for 4 h at room temperature, liver tissue was rinsed in 70% ethanol. Methacarn-fixed tissue samples were then embedded in low-melting point paraffin for preparation of 5 µm sections. Following fixation in formol/calcium (4% formaldehyde in 0.1 M Na-cacodylate pH 7.4, 1% Ca-chloride) for 1.5 h at room temperature, liver tissue was rinsed in wash buffer (0.1 M Na-cacodylate pH 7.4 in water, 1% Ca-chloride). Formol/calcium-fixed tissue samples were then frozen in liquid nitrogen for preparation of 6 µm cryosections. Standard H&E (Haemaluin Carazzi & Eosine) staining was performed on paraffin embedded sections and used to visualize general liver morphology and to determine the degree of hepatocyte ballooning. Standard Oil Red O staining was performed on cryosections and used to determine the degree of steatosis. Immunohistochemical staining for OX-1 was performed in combination with PAS (periodic acid and Schiff reagent)-staining on paraffin embedded sections and used to determine the degree of lobular inflammation. All stainings were performed as described previously [20] and evaluation of liver tissue from 3 animals per group was done according to the standard NAS-scoring method [21]. Hepatocyte ballooning was evaluated in 10 high power fields (HPF) at magnification ×40, whereas steatosis and lobular inflammation were evaluated in 10 HPF at magnification ×20. For each parameter, the median score was obtained per animal, and subsequently for each group. Finally, the overall median NAS score was obtained per group. In case of values >0, Q1 and Q3 values are given.

### Calculation of Liver Weight

Whole liver weight of rats after 10 weeks of CONTROL diet or after 4 weeks of MCD diet was estimated based on previously determined liver weight/body weight ratios for CONTROL and MCD-treated rats, respectively 0,0379 g and 0,0362 g (Boeykens et al., submitted). Further estimation of liver weight directly after PHx in CONTROL or MCD-treated rats was calculated based on the actual weight of the resected liver parts (i.e. estimated liver weight – weight of resected liver part). Evaluation of liver weight in CONTROL, MCD-treated rats and CHEMOTHERAPY-treated rats at the end of the study was done based on the actual weight of the dissected livers.

### Statistical Analysis

A linear mixed effects model was fitted to model the evolution of body weight, serum CHE levels and TSB levels over time, and to study the effect of MCD or CHEMOTHERAPY and/or PHx on this evolution. The fixed part of the model contains an intercept, time-effect, MCD-effect, prior-MCD-effect, PHx-effect, CHEMOTHERAPY-effect, prior-CHEMOTHERAPY-effect and all two- and three-way interactions between them. The repeated measures character of the data is taken into account by including a subject-specific intercept and random slope in the random part of the model. For liver weight, a repeated measures ANOVA with group as between and time as within subjects factor, is used to compare the 4 groups at the 3 time-points. To adjust for multiple testing, false discovery rate correction (FDR) is applied. For NAS scoring, Mann-Whitney U Test was used to compare scores (median (Q1–Q3)) between groups. All analyses were done in SAS 9.2 or IBM SPSS Statistics 20.

## Results

### Study Groups

**Figure 1:**
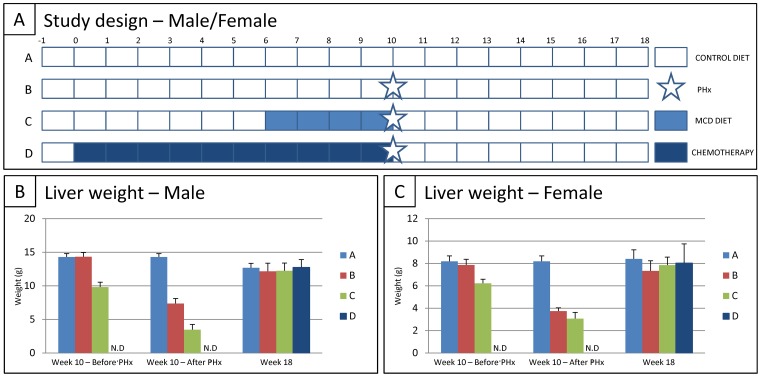
Study design and liver weight following PHx or MCD diet or chemotherapy in combination with PHx. (A) Study design, for both male and female rats. (B/C) Mean liver weight for each experimental group at week 10 before PHx (left panels), at week 10 directly after PHx (middle panels) and at week 18 (end of the study, right panels); for male (B) and female (C). Data are expressed in gram ± standard error.

Health status of CONTROL rats, CONTROL rats+PHx, rats with MCD DIET+PHx and rats with CHEMOTHERAPY+PHx was evaluated based on: (i) liver weight before PHx, after PHx and at the end of the study, (ii) longitudinal measurement of body weight, (iii) longitudinal measurement of serum CHE and TSB levels, and (iv) liver NAS scoring before PHx and at the end of the study. For this experimental set-up, male and female wild type Lewis rats were divided into two times four groups (for both male and female) (figure 1A): (A) control rats fed with control diet for 18 weeks (CONTROL DIET, *n = 10♂ and 10♀*), (B) rats fed with control diet for 18 weeks and subjected to PHx at week 10 (CONTROL DIET+PHx, *n = 10♂ and 10♀*), (C) rats fed with control diet for 6 weeks, followed by MCD diet for 4 weeks, subsequently subjected to PHx and followed by control diet for 8 weeks (MCD DIET+PHx, *n = 10♂ and 10♀*), (D) rats weekly injected subcutaneously with chemotherapy for 10 weeks, subsequently subjected to PHx and followed by control diet for 8 weeks (CHEMO+PHx, *n = 10♂ and 10♀*).

### Effect of MCD Diet on Total Body Weight, Liver Function, Total Liver Weight and Histopathology

**Figure 2:**
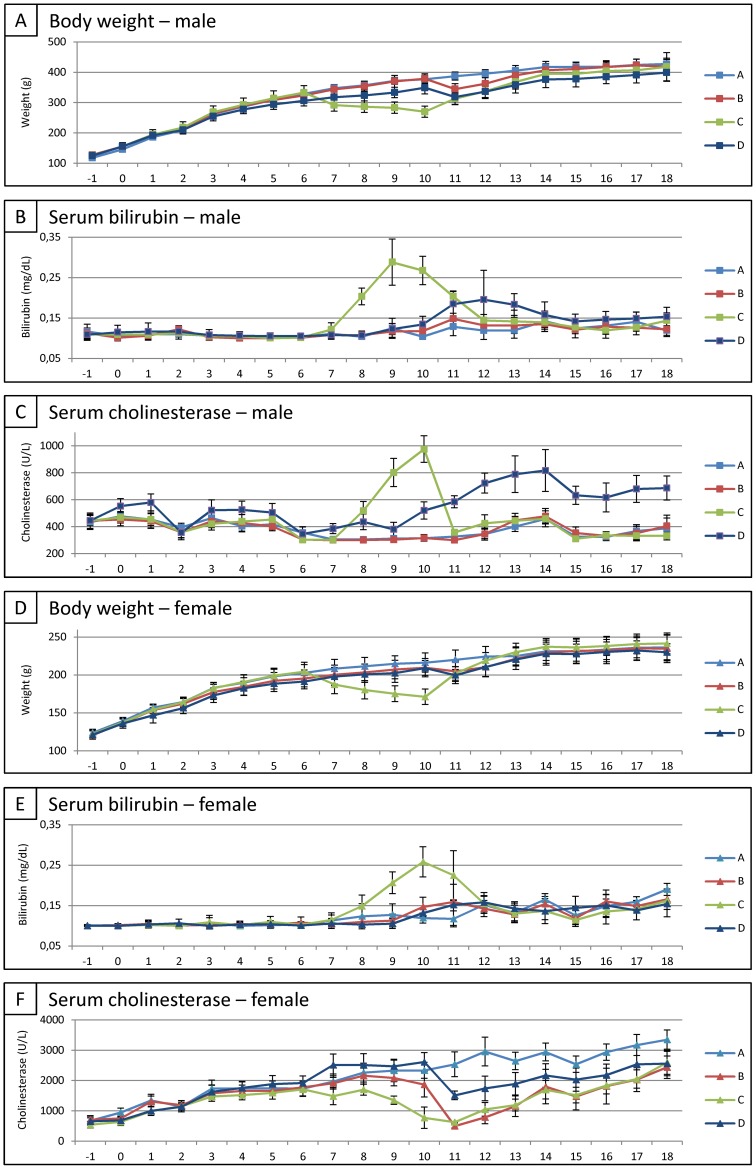
Recovery assessment following partial hepatectomy or MCD diet or chemotherapy in combination with PHx. (A/D) Evolution of body weight of male/female rats for each experimental group. Data are expressed in gram ± standard error. (B/E) Evolution of total serum bilirubin (TSB) of male/female rats for each experimental group. Data are expressed in milligram per deciliter ± standard error. (C/F) Evolution of serum cholinesterase (CHE) level of male/female rats for each experimental group. Data are expressed in units per liter ± standard error.

**Figure 3:**
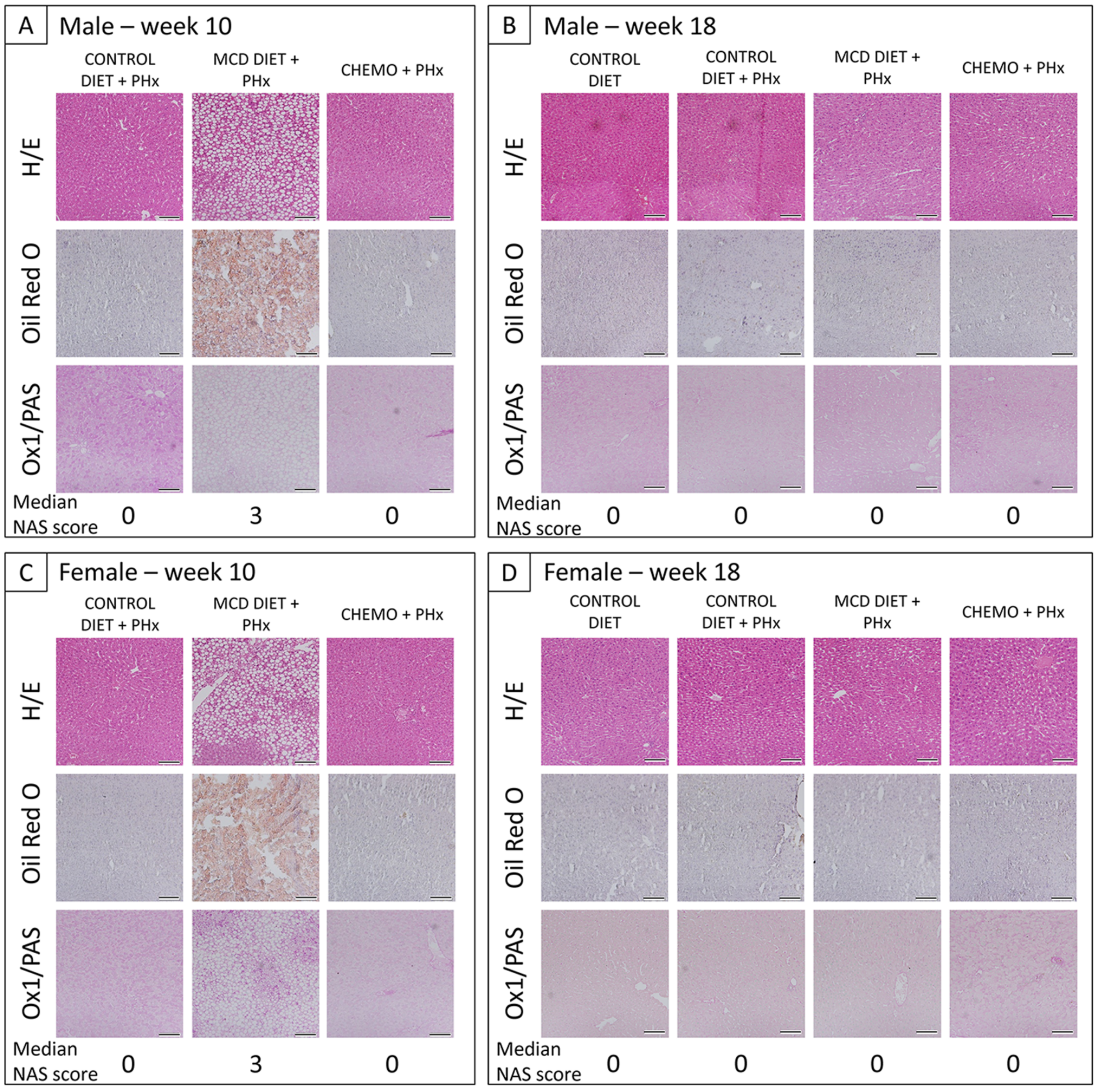
Histological analysis of liver tissue following PHx or MCD diet or chemotherapy in combination with PHx. Haematoxylin-eosin (H/E) staining (upper panel), Oil Red O staining (middle panel) and PAS/OX-1 staining (lower panel) (A/C) for each experimental group (which is subjected to PHx) of male/female rats at week 10, (B/D) for each experimental group of male/female rats at week 18. Representative images were chosen out of 3 rats analyzed per experimental group. Scale bars indicate 100 µm.

At first, body weight of rats in all experimental groups, i.e. CONTROL DIET ± PHx and MCD DIET+PHx, was measured weekly during the whole experimental set-up as a non-invasive parameter to monitor general animal health. As shown in figure 2A and 2D, for both males and females, a significant decrease in body weight is observed after 4 weeks of MCD diet (CONTROL DIET ± PHx (GROUP A+B) vs. MCD DIET+PHx (GROUP C); 378,1 g±14,4 g vs. 269,9 g±18,2 g for male rats (p<0,0001) and 213,1 g±12,5 g vs. 171,3 g±10,2 g for female rats (p<0,0001)). During the same time-period, both serum CHE and TSB levels were determined weekly. A significant increase in total serum bilirubin (TSB) levels is observed after 4 weeks of MCD diet, for both male and female rats (CONTROL DIET ± PHx (GROUP A+B) vs. MCD DIET+PHx (GROUP C); 0,111 mg/dl±0,016 mg/dl vs. 0,268 mg/dl±0,035 mg/dl for male rats (p = 0,0051) and 0,133 mg/dl±0,023 mg/dl vs. 0,258 mg/dl±0,037 mg/dl for female rats (p<0,0001)), as shown in figure 2B and 2E. For serum CHE levels, a significant increase is observed after 4 weeks of MCD diet in male rats (CONTROL DIET ± PHx (GROUP A+B) vs. MCD DIET+PHx (GROUP C); 315 U/l±22 U/l vs. 975 U/l±99 U/l (p<0,0001). In contrast, female rats display a significant decrease in serum CHE levels after 4 weeks of MCD diet (CONTROL DIET ± PHx (GROUP A+B) vs. MCD DIET+PHx (GROUP C); 2114 U/l±408 U/l vs. 771 U/l±352 U/l (p<0,0001) (figure 1F). Next, rats fed with control diet (GROUP B) and MCD diet (GROUP C) underwent a PHx. Based on the actual weight of the resected liver parts and the estimated whole liver weight before PHx (based on previously determined liver/body weight ratios for control and MCD diet fed rats), the whole liver weight before and after PHx was estimated for CONTROL DIET (GROUP A), CONTROL DIET+PHx (GROUP B) and MCD DIET+PHx (GROUP C). As shown in figure 1B and 1C (left panels), for both male and female, a significant decrease in liver weight is observed after 4 weeks of MCD diet (CONTROL DIET ± PHx (GROUP A+B) vs. MCD DIET+PHx (GROUP C); 14,29 g±0,50 g vs. 9,82 g±0,70 g for male rats; 8,19 g±0,48 g vs. 6,22 g±0,36 g for female rats; for both p<0.0001). Subsequently, a PHx further decreased the total liver weight, as shown in figure 1B and 1C (middle panels). Finally, resected liver parts from control rats (GROUP B) and MCD diet-fed rats (GROUP C) underwent histological analyses to determine liver injury. Following H&E, Oil Red O and Ox1/PAS staining, evaluation of tissue sections was performed according to the NAS-scoring method, based on the degree of hepatocyte ballooning, steatosis, and lobular inflammation (for each group (B and C) and both for male and female, 3 livers were analyzed). As shown by the representative images in figure 3 and the provided median NAS scoring (for detailed NAS scoring see table S1), similar results were obtained for male and female rats. Following 4 weeks of MCD diet, livers display significant steatosis (figure 3A/C middle panel, MCD DIET+PHx, median NAS score 3), but no hepatocyte ballooning or lobular inflammation, as compared to livers from control rats (figure 3A/C left panel, CONTROL DIET+PHx, median NAS score 0), indicating successful induction of steatosis by the MCD diet.

### Recovery of Total Body Weight, Liver Function, Total Liver Weight and Histopathology Following PHx of Steatotic Livers

Following PHx and after cessation of the MCD diet, body weight immediately increased in both male and female rats (figure 2A and 2D). However, while female rats equaled total body weight of control female rats after 2 weeks, male rats did not equal total body weight of control male rats at the end of the study (CONTROL DIET (GROUP A) vs. MCD DIET+PHx (GROUP C); 427,3±19,5 vs. 418,1±46,3; p<0,0001). No significant effect on total body weight was observed following PHx in rats fed with control diet (GROUP B). Additionally, as shown in figure 2B and 2E, upon cessation of MCD diet after PHx (GROUP C), TSB levels immediately decreased equaling the TSB levels of control rats (GROUP A) after 4 weeks in male rats and 2 weeks in female rats. No effect of a PHx on serum TSB levels was observed for both male and female control rats (GROUP B). As shown above, serum CHE levels display opposite patterns in both male and female rats. CHE levels of male rats immediately decrease (GROUP C), equaling the CHE levels of control rats (GROUP A) after 1 week. Notably, a PHx alone in control male rats did not display an effect on CHE levels (GROUP B). In contrast, CHE levels of female rats immediately increase without equaling the CHE levels of control rats at the end of the study (CONTROL DIET (GROUP A) vs. MCD DIET+PHx (GROUP C); 3347±317 vs. 2596±394; p<0,0001) Notably, and in contrast to male rats, in female rats a PHx displays a significant effect on serum CHE levels (GROUP B). Serum CHE levels decrease immediately after PHx, start increasing one week later, however CHE levels do not equal levels of control rats at the end of the observation period (CONTROL DIET (GROUP A) vs. CONTROL DIET+PHx (GROUP B); 3347±317 vs. 2439±369; p<0,0001). At the end of the study, control and regenerated livers were resected, weighted and processed for histology. Results given in figure 1B and 1C (right panels) clearly indicate that liver weight returns to control levels (GROUP A; 12,68 g±0,67 g for male and 8,40 g±0,81 g for female) for both male and female rats after CONTROL DIET+PHx (GROUP B, 12,16 g±1,20 g for male rats and 7,36 g±0,90 g for female rats) and after MCD DIET+PHx (GROUP C, 12,24 g±1,16 g for male rats and 7,85 g±0,71 g for female rats) following PHx and 8 weeks of CONTROL DIET. Additionally, for both male and female rats, histological analysis revealed that after PHx and 8 weeks on CONTROL diet, liver tissue regenerated both in CONTROL DIET+PHx and MCD DIET+PHx groups, without significant signs of steatosis, lobular inflammation or hepatocyte ballooning (figure 3B/D middle right panel, MCD DIET, NAS score 0, table S1), as compared to livers from control rats of the same age.

### Effect of FOLFOX Chemotherapy on Total Body Weight, Liver Function and Histopathology

At first, body weight of rats in all experimental groups, i.e. CONTROL DIET ± PHx and CHEMO+PHx, was measured weekly during the whole experimental set-up as a non-invasive parameter to monitor general animal health. As shown in figure 2A, a slight decrease in body weight is observed after 10 weeks of CHEMOTHERAPY in male rats (CONTROL DIET ± PHx (GROUP A+B) vs. CHEMO+PHx (GROUP D); 378,1 g±14,4 g vs. 349,3 g±20,5 g (p<0,0001)). In contrast, no differences could be observed following 10 weeks of CHEMOTHERAPY in female rats (figure 2D). During the same time-period, both serum CHE and TSB levels were determined weekly. No differences could be observed on TSB and CHE levels of rats treated with CHEMOTHERAPY for 10 weeks compared to control rats, for both male and female rats, as shown in figure 2B/C and 2E/F respectively. Next, control rats (as described above) and rats treated with CHEMOTHERAPY underwent a PHx. Subsequently, resected liver parts from rats treated with CHEMOTHERAPY underwent histological analyses to determine liver injury. As shown by the representative images in figure 3 and the provided NAS scoring, no significant signs of hepatocyte ballooning, steatosis, and lobular inflammation was observed in both male and female rats (figure 3A/C right panel, MCD DIET+PHx, median NAS score 0, table S1).

### Recovery of Total Body Weight, Liver Function, Total Liver Weight and Histopathology Following PHx of FOLFOX Chemotherapy-treated Livers

Following CHEMOTHERAPY and PHx, body weight of male rats immediately increased, however without equaling the total body weight of control rats at the end of the study (CONTROL DIET (GROUP A) vs. CHEMO+PHx (GROUP D); 427,3±19,5 vs. 399,1±28,8; p<0,0001) (figure 2A). In female CHEMOTHERAPY treated rats (GROUP D), no significant effect of a PHx on total body weight was observed (figure 2D). Additionally, as shown in figure 2B, following PHx in CHEMOTHERAPY-treated rats TSB levels only slightly increase in male, but not in female rats. However TSB levels quickly recover to those of control rats (GROUP A) at week 17. In contrast, serum CHE levels again display opposite patterns in both male and female rats after PHx, as shown in figure 2C and 2F. In female rats, CHE levels significantly decrease directly following PHx (p<0,0001). However, CHE levels immediately start recovering without equaling those of control rats at the end of the study (CONTROL DIET (GROUP A) vs. CHEMO+PHx (GROUP D); 3169 U/l±346 U/l vs. 2537 U/l±557 U/l; p<0,0001). Surprisingly CHE levels of male rats increase after PHx on livers from chemotherapy-treated rats, without returning to CHE levels of control rats at the end of the experiment (CONTROL DIET (GROUP A) vs. CHEMO+PHx (GROUP D); 367 U/l±47 U/l vs. 680 U/l±100 U/l, p<0,0001). At the end of the study, control and regenerated livers were resected, weighed and processed for histology. Results given in figure 1B and 1C (right panels) clearly indicate that liver weight shows no significant differences with control levels (CONTROL DIET; 12,68 g±0,67 g for male and 8,40 g±0,81 g for female) for both male and female rats after CHEMO+PHx (12,75 g±1,16 g for male rats and 8,03 g±1,71 g for female rats) following PHx and 8 weeks of CONTROL DIET. Additionally, for both male and female rats, histological analysis revealed that after PHx and 8 weeks on CONTROL diet, liver tissue regenerated in CHEMO+PHx groups, without significant signs of steatosis, lobular inflammation or hepatocyte ballooning (figure 3B/D right panels, CHEMO+PHx, NAS score 0, table S1), as compared to livers from control rats of the same age.

## Discussion

In this study, we aimed to evaluate liver serum parameter levels as long-term non-invasive markers of liver function in male and female rats following liver injury, both before and after partial hepatectomy (PHx). Several significant differences could be noted dependent on either the disease model and/or the gender.

After 4 weeks on MCD diet (steatosis model) followed by PHx, significant differences in body weight and liver weight were observed in both male and female rats. However, all levels equaled control levels at the end of the observation period (except for body weight of male rats). In our search for suitable serum parameters to longitudinally monitor rat liver function, we analyzed AP, GOT, GPT, Cr, CHE and TSB levels. No significant differences in both male and female rats were observed in serum AP, GOT, GPT, Cr after 4 weeks of MCD diet (see figure S1). In contrast, MCD diet induced significant alterations in serum CHE and TSB levels in both genders. Regardless gender, TSB levels increased significantly after 4 weeks of MCD diet, but equaled control levels at the end of the observation period. More remarkably, opposite effects in male and female rats were observed on serum CHE levels, which did not equal control levels at the end of the study in female rats. Although it is known that CHE activity of mature female rats is three to five times higher compared to mature male rats [22], further research is needed to better understand opposite reaction of CHE levels in male and female rats after MCD diet. Although we observed complete recovery of body weight, liver weight and TSB levels after PHx and cessation of the MCD diet, according serum CHE levels we imply that liver function did not recover completely in female rats. Therefore, despite the frequent use of imaging techniques, histological analyses and determination of the body/liver weight ratio for evaluation of liver recovery [23], [24], we here demonstrate that longitudinal assessment of liver function is imperative to identify long-term functional deficits or impaired/improved regeneration. In this regard, female rats are preferential for further regeneration studies and future cell therapy studies. The significant longitudinal differences in serum CHE levels, which after a recovery period of 8 weeks do not equal those of control rats, allows us to study improved recovery both in time and in function. Due to the one-week recovery period of serum CHE levels after PHx and cessation of MCD diet in male rats, these animals are not favored for longitudinal evaluation of therapeutic interventions.

Following 10 weeks of FOLFOX treatment followed by PHx, no significant (or only minor) effects on body weight, liver weight and TSB levels were observed. Moreover, histological analyses revealed no changes in liver architecture, i.e. steatosis, hepatocyte ballooning or inflammation. As for the steatosis model, opposite effects on serum CHE levels were observed in male and female rats, where in male rats a long-term increase in CHE levels is observed as compared to a long-term decrease of CHE levels in female rats. As described above, further research will need to address the nature of this gender-specific difference. On the other hand, despite the fact that the association between FOLFOX chemotherapy and liver injury is frequently described in patients [7], [8], [9], [11], these observations have been difficult to reproduce in rodents [25], [26]. Our study thus indicates that, even in the absence of histological evidence for liver injury, significant alterations in liver function can be observed as a consequence of PHx following chemotherapy. However, the impact of these alterations on general health and/or liver function will need further investigation. Of note, preceding optimization studies to determine the optimal dose of FOLFOX chemotherapy before PHx revealed that a higher dose of FOLFOX treatment (1.5X current dose) also did not result in significant histological alterations, but resulted in significant mortality (50%) after PHx (data not shown). Thereby again underscoring the fact that histological analyses alone cannot provide an accurate estimation of liver function in preclinical rodent models. Nevertheless, given the observation of long-term alterations of serum CHE levels in both male and female rats, further studies regarding therapeutic intervention after chemotherapy+PHx can be performed in either sex and might reveal further gender-specific differences in liver function and/or regeneration.

### Conclusions

The results presented in this study strongly encourage the inclusion of longitudinal measurements of biochemical parameters to be essential for evaluation of liver injury and liver recovery in rat models of PHx following liver injury. The latter is underscored by the observation that long-term alterations in CHE levels can be detected, even though histological analyses on liver tissue indicate absence of injury and/or full regeneration. Moreover, specific attention should be given to the choice of male or female rats for these studies, as opposite effects in CHE levels are clearly observed.

## Supporting Information

Figure S1 **Serum parameter levels following control diet (control), methionine/choline deficient diet (MCD) or chemotherapy treatment (CHEMO) in rat.** Serum parameter levels of male (♂) and female (♀) rats of 5 pooled serum samples of each experimental group (i.e. after 4 weeks of control diet (control), 4 weeks of MCD diet (MCD) of after 10 weeks of chemotherapy treatment (CHEMO)). (A) Serum bilirubin. Data are expressed in milligram per deciliter ± analytical error. (B) Serum cholinesterase. Data are expressed in units per liter ± analytical error. (C) Serum GPT. Data are expressed in units per liter ± analytical error. (D) Serum alkaline phosphatase. Data are expressed in units per liter ± analytical error. (E) Serum GOT. Data are expressed in units per liter ± analytical error. (F) Serum creatinin. Data are expressed in milligram per deciliter ± analytical error.
(TIF)

Table S1 **Calculation of the median NAS score for the different experimental groups at selected time points.**
(DOCX)
