# Supplementary figures and images for: Biochemical Parameters for Longitudinal Monitoring of Liver Function in Rat Models of Partial Hepatectomy Following Liver Injury
Source: PLoS One. 2013 Jun 18;8(6):e66383. doi: 10.1371/journal.pone.0066383 (PMC3688924; doi:10.1371/journal.pone.0066383)

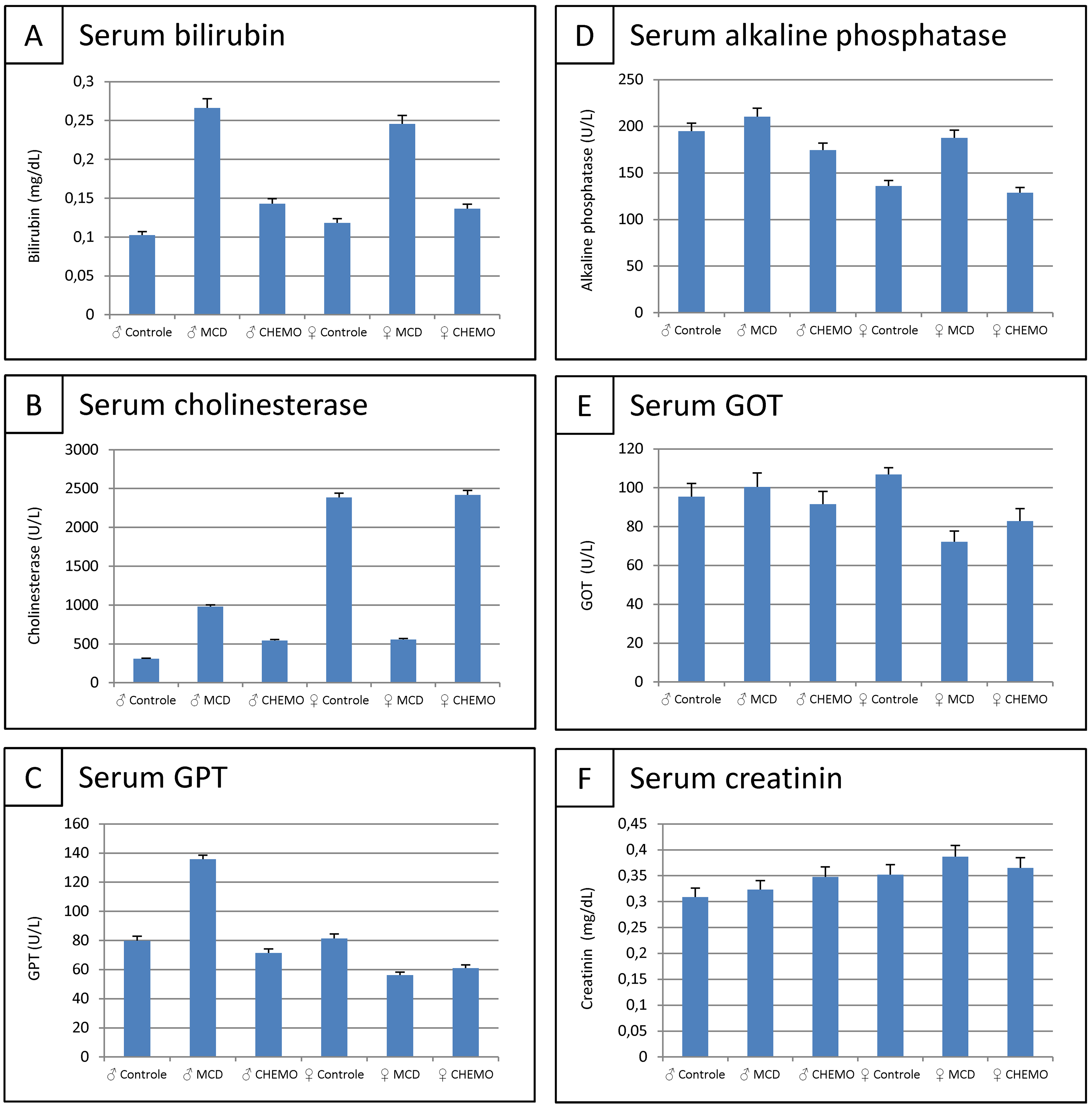

Supplement: Figure S1 — Serum parameter levels following control diet (control), methionine/choline deficient diet (MCD) or chemotherapy treatment (CHEMO) in rat. Serum parameter levels of male (♂) and female (♀) rats of 5 pooled serum samples of each experimental group (i.e. after 4 weeks of control diet (control), 4 weeks of MCD diet (MCD) of after 10 weeks of chemotherapy treatment (CHEMO)). (A) Serum bilirubin. Data are expressed in milligram per deciliter ± analytical error. (B) Serum cholinesterase. Data are expressed in units per liter ± analytical error. (C) Serum GPT. Data are expressed in units per liter ± analytical error. (D) Serum alkaline phosphatase. Data are expressed in units per liter ± analytical error. (E) Serum GOT. Data are expressed in units per liter ± analytical error. (F) Serum creatinin. Data are expressed in milligram per deciliter ± analytical error. (TIF) [file pone.0066383.s001.tif]
