# Supplementary material for: Biochemical Parameters for Longitudinal Monitoring of Liver Function in Rat Models of Partial Hepatectomy Following Liver Injury
Source: PLoS One. 2013 Jun 18;8(6):e66383. doi: 10.1371/journal.pone.0066383 (PMC3688924; doi:10.1371/journal.pone.0066383)
Supplement: Table S1 — Calculation of the median NAS score for the different experimental groups at selected time points. (DOCX) [file pone.0066383.s002.docx]

**Table S1.** **Calculation of the median NAS score for the different experimental groups at selected time points.**

| **Gender** | **Group** | **Week** | **Median score for ballooning** | **Median score for steatosis (Q1-Q3)** | **Median score for lobular inflammation** | **Median NAS-score (Q1-Q3)** |
| --- | --- | --- | --- | --- | --- | --- |
| ♂ | A | 10 | n.a. | n.a. | n.a. | **n.a.** |
|  | B | 10 | 0 | 0 | 0 | **0** |
|  | C | 10 | 0 | 3 (3-3) | 0 | **3 (3-3)** |
|  | D | 10 | 0 | 0 | 0 | **0** |
|  | A | 18 | 0 | 0 | 0 | **0** |
|  | B | 18 | 0 | 0 | 0 | **0** |
|  | C | 18 | 0 | 0 | 0 | **0** |
|  | D | 18 | 0 | 0 | 0 | **0** |
| ♀ | A | 10 | n.a. | n.a. | n.a. | **n.a.** |
|  | B | 10 | 0 | 0 | 0 | **0** |
|  | C | 10 | 0 | 3 (3-3) | 0 | **3 (3-3)** |
|  | D | 10 | 0 | 0 | 0 | **0** |
|  | A | 18 | 0 | 0 | 0 | **0** |
|  | B | 18 | 0 | 0 | 0 | **0** |
|  | C | 18 | 0 | 0 | 0 | **0** |
|  | D | 18 | 0 | 0 | 0 | **0** |
